# Updated Methods for the Production of *Toxorhynchites rutilus septentrionalis* (Diptera, Culicidae) for Use as Biocontrol Agent Against Container Breeding Pest Mosquitoes in Harris County, Texas

**DOI:** 10.1093/jisesa/iez011

**Published:** 2019-03-07

**Authors:** Anita Schiller, Mary Allen, Jewel Coffey, Arielle Fike, Franklin Carballo

**Affiliations:** Biological Control Mosquito Initiative, DRAC/MAP Harris County Precinct, Commissioner Cagle, Louetta Road, Spring TX

**Keywords:** mass-rearing technique, Toxorhynchites, mosquito predator, mosquito assassin, biological control

## Abstract

The complex biologies of pestiferous mosquito species complicate the development of a single, across the board effective control measure. The use of traditional biological control through predators, parasitoids, and microbes, though part of a multimodal, integrated pest management approach, is scant in current standard mosquito control operations. At this time, traditional, whole organism mosquito biocontrol agents are not commercially available, and if their integration into a release program is desired, they must be developed in-house. The aim of our program was to incorporate releasing natural enemies to disrupt and suppress the target pest mosquito’s population by matching the agent with the target’s biology, before or concurrent to pesticide use. A current focus is suppressing peridomestic, container breeders of high medical significance, such as *Aedes aegypti* (Linnaeus), (Diptera, Culicidae) and *Aedes albopictus* (Skuse) (Diptera, Culicidae), through larval reduction with augmentative releases of laboratory-reared, native mosquito assassins *Toxorhynchites rutilus septentrionalis* (Dyar & Knab). When raised in communal settings, *Tx. rutilus’* aggressive feeding behavior and cannibalistic tendencies require an extreme loss tolerance in adult production rates. In addition, offering prey mosquitoes exclusively as *Tx. rutilus’* juvenile food extends larval development by multiple days. While this may be desirous in the wild, it proves inefficient during production. Here, we provide an individual rearing method as well as an alternative diet protocol, which maximizes the adult yield while achieving quick development.

Quite ubiquitous, both *Aedes aegypti* (L.) and *Aedes albopictus* (Skuse) occur in Harris County, Texas, where they are aggressive human biters and competent vectors of emergent, burdensome tropical disease viruses including Dengue, Zika, Chikungunya, and Yellow Fever. Their preferred larval habitat is primarily found close to human habitation, yet individual larval sites are often cryptic and exploitive of neglected and unsanitary conditions, thus typically difficult to treat with conventional larvicides. Flower pots, clogged gutters, tree holes, children’s plastic yard toys, kiddie pools, pet watering bowls, and unused vehicle tires are examples of peridomestic *Aedes* mosquito breeding sites. Extirpated from much of the heavily urbanized metropolis of Houston and surrounding Harris County, Texas, the native mosquito *Toxorhynchites rutilus septentrionalis* (Dyar and Knab) is a proven effective larval mosquito predator (Fig. 1). Therefore, it is considered a beneficial organism worth releasing to reduce localized container-breeding mosquito populations naturally. *Toxorhynchites* spp. show a strong preference for ovipositioning into the same containers as *Ae. aegypti* and *Ae. albopictus* and a single *Tx. rutilus* larva are reported to eliminate a few hundred to 5,000 prey larvae (Focks 2007). Though less than during its quicker summer development, the killing of other macroinvertebrates within the same container continues during winter diapause. In the absence of mosquito prey, the predator will also feed on chironomid larvae, aquatic worms, and even nonmotile detritus (Collins and Blackwell 2000) in its fourth and last larval instar. The entire nutritional requirement for *Toxorhynchites* spp. oogenesis is met during its larval form; adults are incapable of taking a bloodmeal and take only floral nectar as nourishment. Not much is known about adult *Toxorhynchites* spp. floral preferences, such as choice of flower shapes or colors. It is also not known if they play a vital role in pollination ecology, or if they are generalist flower visitors. While their use as a biocontrol agent is in the larval form, all life stages can be released. The integrated model of releasing gravid adult *Toxorhynchites amboinensis* (Doleschall) females into an *Ae. aegypti* hotspot post-ultra-low-volume adulticiding (ULV) with Malathion was tested and demonstrated to be a more effective approach than either ULV Malathion pesticide application or *Tx. amboinensis* releases alone (Collins and Blackwell 2000). The DRAC/MAP mosquito assassin efficacy field study uses the native *Tx. rutilus* instead. The native mosquito assassin is known to be difficult to rear in captivity as high cannibalism rates complicate their husbandry and production. Several attempts to rear *Tx. rutilus* following published communal rearing methods (Crans and Slaff 1977, Focks and Boston 1979) yielded unsatisfactory development times and inadequate quantities of releasable adults. However, the difficulty in rearing is offset by advantageous traits: its ability of *Tx. rutilus* to become locally established, withstand local environmental extremes, winter diapause, and early seasonal flight activity. Therefore, we devised an alternative production method that keeps larvae from predating on one another and utilizes foods that are easier to rear and feed than prey mosquitoes.

**Fig. 1.**
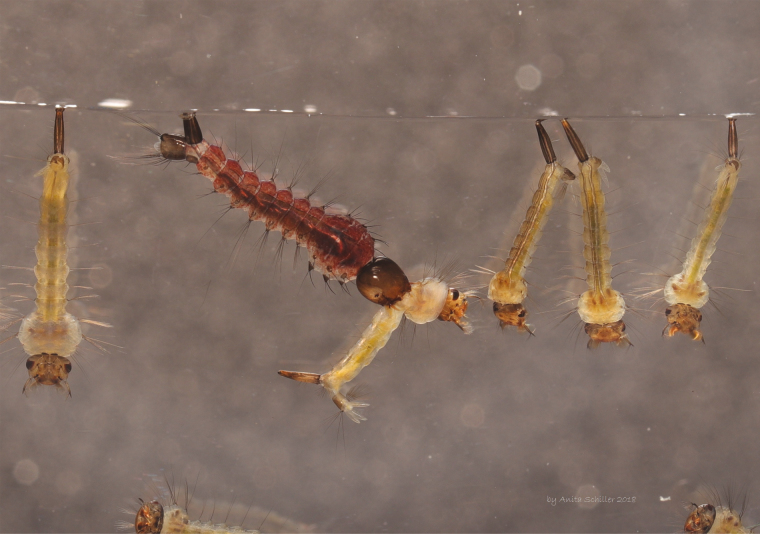
*Toxorhynchites rutilus* larva consuming a *Culex* sp., pest mosquito larva.

## Materials and Methods

### Colony Origins

Production took place from a domesticated *Tx. rutilus* colony that originated in 2013 from 1,042 locally field collected eggs and reared ex situ. Only animals that completed larval development from egg to pupa in under 17 d were selected to establish the colony. An exception was made during the winter diapause when, in order to keep the colony thriving, selected development age was pushed to 20 d. Additionally, a liquid antibiotic, PSF (Penicillin, Streptomycin, and Amphotericin B), was added to the rearing waters and sucrose feeders (but not fructose) to eliminate chronic and acute internal infections. At the time of the 2017 efficacy field study, the colony was at approximately successive generation 50 and PSF was administered only to countermeasure colony die off.

### Insectary Conditions

The *Tx. rutilus* insectary rooms are maintained at 80–85°F/26–29°C during the day and 70–75°F/21–24°C at night with a diel of 14:10 (L:D) h. Humidity is increased to 85–90% early morning at 4 a.m., again between 6 and 8 a.m. and in the afternoon between 4 and 6 p.m. via a fog unit (Hydrofogger.com). Humidity levels are allowed to drop to 40% in between foggings.

### Cage

**Fig. 2.**
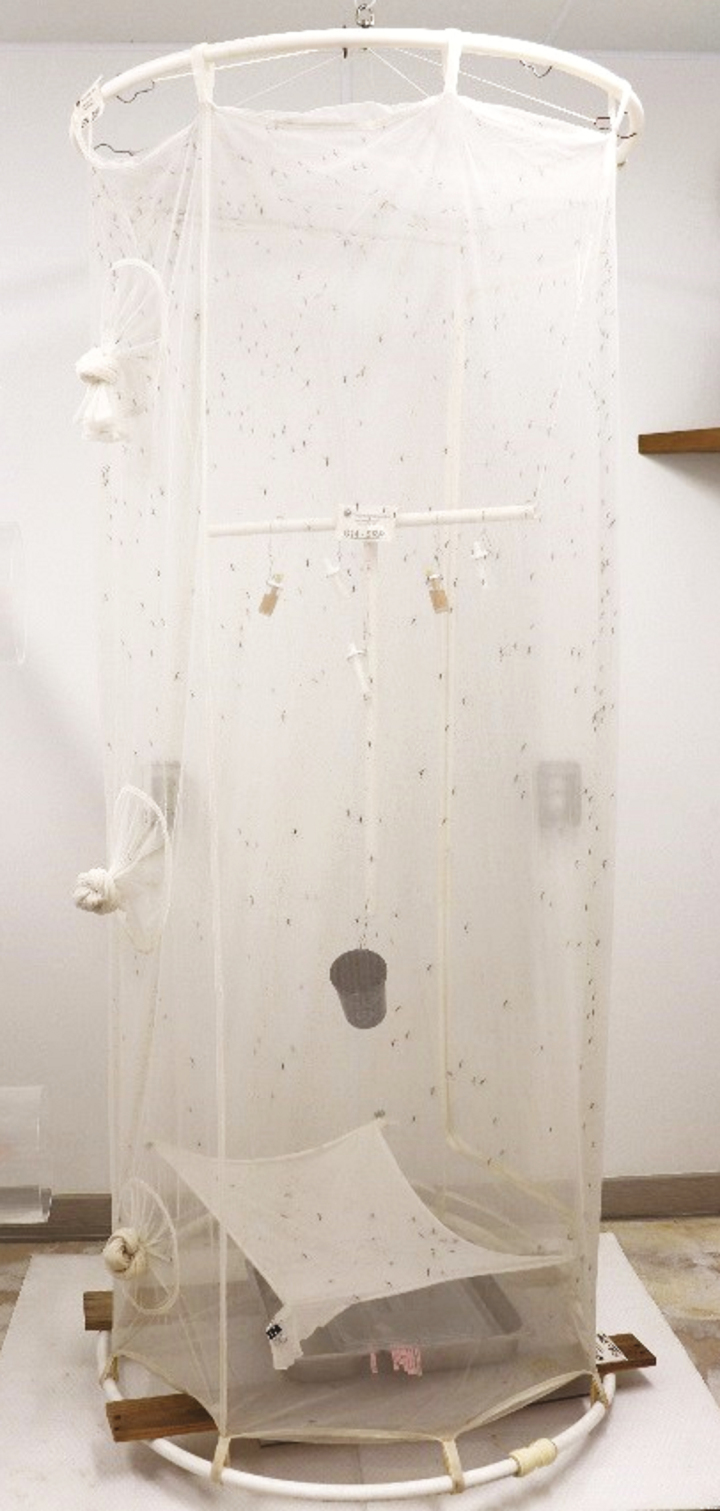
The *Tx. rutilus* cage with hanging sugar feeders, water, and oviposition cup. In the bottom of the cage is the pupa emergence pan and screen cover.

**Fig. 3.**
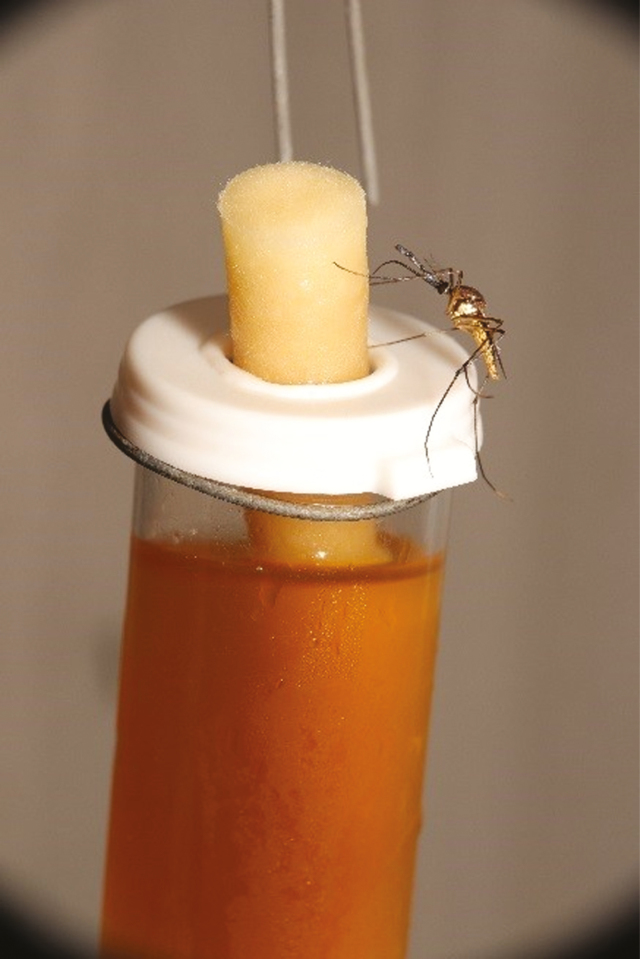
Inside the adult *Tx. rutilus* cage, a close up of a honey feeder with an adult female *Tx. rutilus* feeding off the honey soaked dental wick.

The production colony is housed in custom cylindrical 180 × 90 cm cages completely made of 0.5- × 0.3-mm mesh (BioQuip.com #1451NS12) and populated with an average of 650 mixed sex adult *Tx. rutilus*. The hanging frames are made of bendable pipe, hung from the ceiling with swivel hooks for 360° access, and are attached to the cage exterior with Velcro loops (Fig. 2). The entire mesh cage can be laundered in a washing machine. Four 15-ml vials are hung in the cage, two with a 10% sucrose solution and two with a 50% honey solution, along with one 30-ml vial of 100% fresh water, all outfitted with a cotton dental wick, and are replaced twice weekly (Fig. 3).

### Egg Collection

**Fig. 4.**
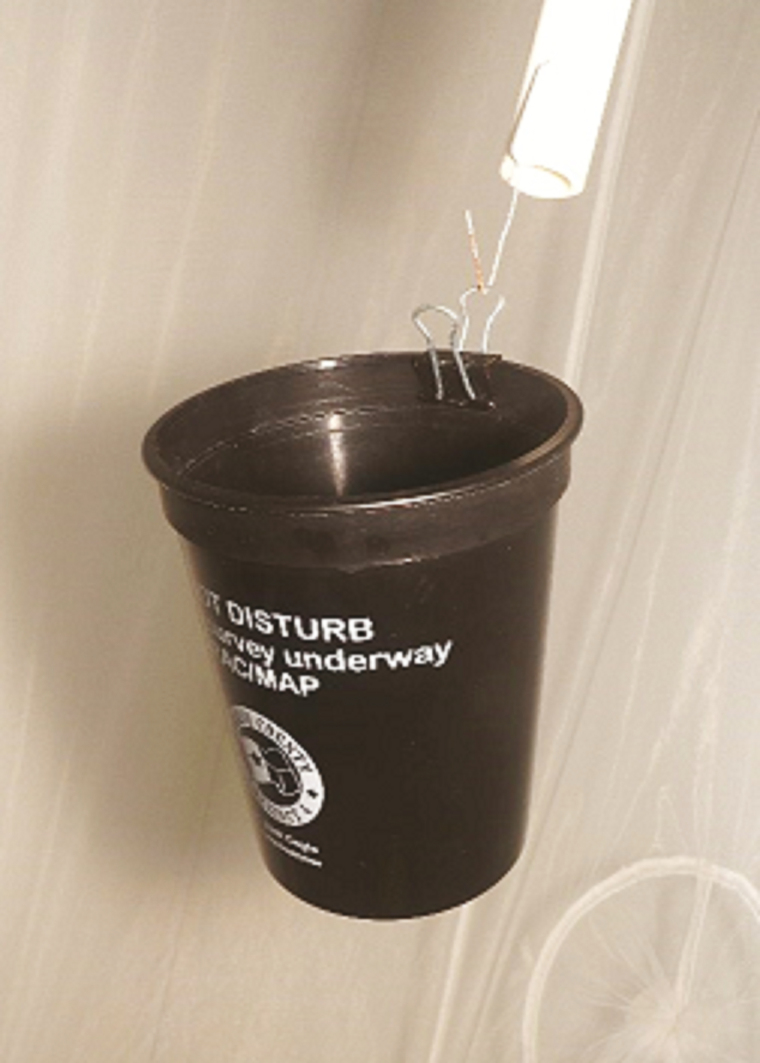
Black oviposition cup hanging inside the adult *Tx. rutilus* cage.

Eggs are collected by hanging a 473-ml black plastic cup (Fig. 4) inside the cage for 24 h. The cup is partially filled with 120 ml of reverse osmosis (RO) water, lightly infused with an aquaculture supplement made with Melafix (active ingredient: *Melaleuca alternifolia*, Myrtaceae). The *Melaleuca* fragrance is similar to that given off by the native sweetgum tree (*Liquidambar styraciflua*), black gum (*Nyssa sylvatica*), and red bay (*Persia borbonia*). These tree species appear to be preferential *Tx. rutilus* oviposition trees, occur in the forested areas of southeast Texas, and are high in essential oils in their bark and leaves. Furthermore, *Melaleuca* infusion inhibits microbial and fungal growth. *Toxorhynchites* spp. females oviposit during a looping flight and release single eggs by tossing them one by one onto the water. Only when crowded or when ambient temperatures are above 90°F/32.2°C will the females land on the water surface to oviposit. Newly laid eggs are matte whitish colored, somewhat oval shaped, clump together, and darken to a light gray during embryonation. The dimpled egg chorion repels water; thus, any breeze or disturbance can bounce the eggs out of the vessel. Eggs blown from the water desiccate and are doomed. The black cup containing the entire egg batch is removed no later than 24 h. Using a simple scoop made of 30-µm diameter mesh, eggs are floated onto fresh RO water and counted via an image taken by an iOS cell counter App (Promega Corporation 2015). To minimize loss to cannibalization, large egg batches are divided into smaller quantities and separate containers so that the hatchlings will have at least 2 cm^2^ of surface area each (Table 1). Calculating staff work schedules and taking prey culture timing into account, egg batches are checked for eclosion from 38 h at 85°F/29.4°C (quicker) or 44 h when kept at 80°F/26.6°C, respectively (Table 2). To reduce loss from cannibalism, once hatch commences multiple inspections for new hatchlings are necessary for an additional 36–48 h. Hatchlings, whose head capsule shows sclerotization (Crans and Slaff 1977), are moved to their individual rearing cells via a clear 3-ml pipette. Four-day-old unhatched eggs are considered infertile and discarded.

**Table 1.** Surface area ratio egg/pupae

Restaurant style container size	No. of Eggs	Surface area/egg (cm^2^)	No. of pupae	Surface area/pupae (cm^2^)
Full	700	2.1	250	5.8
1/2	350	2	125	5.7
1/3	175	2.5	75	5.9
8-oz bowl	50	2	25	3.8
2 oz	10	3	5	5.2
1 oz	5	2.5	1	12.6

**Table 2.** *Toxorhynchites rutilus* egg eclosion at differing temperatures, ex situ

Trial	Hours to eclosion at 80°F/26.6°C	Hours to eclosion at 85°F/29.4°C	Hours to eclosion at 90°/32.2°C
1	42	38	X
2	43	39	X
3	45	38	X
4	44	37	X
Mean	**43.5**	**38**	No hatch

### Larval Rearing Trays

**Fig. 5.**
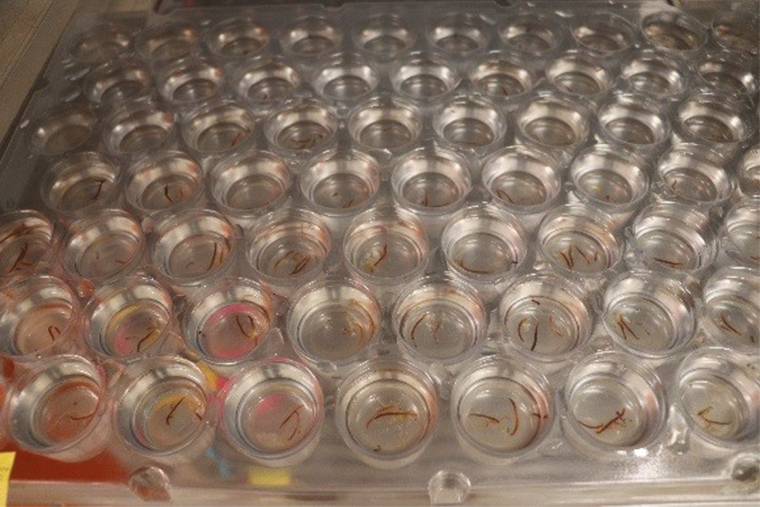
Isolation trays where *Tx. rutilus* are placed individually in separate wells to prevent cannibalism.

**Fig. 6.**
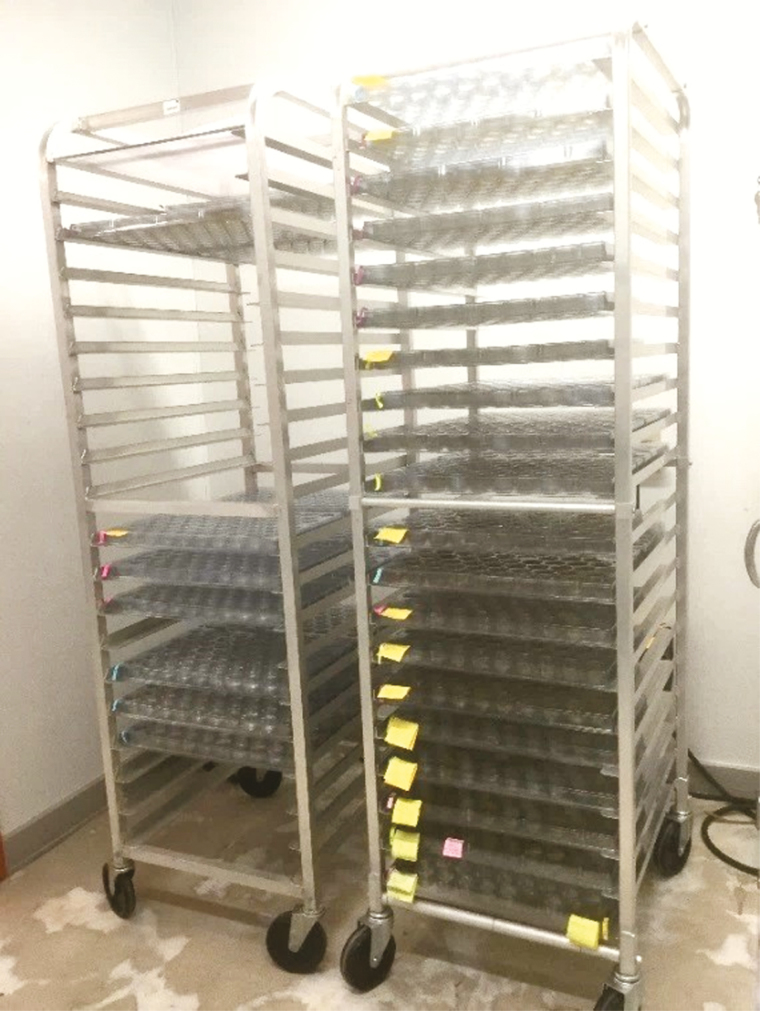
Standard aluminum restaurant-style bun rack holding the custom cell trays.

Our trays are custom cell trays (Fig. 5) made of clear, 0.029-ml gauge, recycled polyethylene terephthalate and stacked onto a standard aluminum restaurant-style bun rack (Fig. 6).

Each cell is prefilled with 10 ml of RO water, and if time allows, it is preloaded with *Panagrellus* worms. To ensure the hatchlings’ exposure to the settled food, initial water levels are kept quite shallow. Subsequent feedings and water additions will increase water volume to the maximum 25 ml at L4 stage. In our experience, the use of sterile distilled or deionized water is not advisable; however, the addition of nitrifying beneficial bacteria (aquaculture supplement) into deionized water increases larval survival by supporting waste conversion.

### Food

Our feeding protocol includes three different types of food, of which two are cultured in-house, whereas one is store bought and kept frozen until use. We compared this varied food protocol against feeding exclusively live prey mosquitoes and determined that the time and labor required to rear, colonize, and blood feed the necessary quantity of prey mosquitoes are much greater when compared with the alternative diet. Feeding live prey mosquitoes is inferior in several ways: 1) *Tx. rutilus* larval development is slower when fed solely prey mosquitoes; 2) tasks associated with maintaining a dozen or more live blood feeding mosquito cages to culture *Tx. rutilus* larval food take multiple staff; 3) balancing the feeder mosquito’s life cycle for size and development days to be available at the proper time for multiaged batches is resource prohibitive; 4) Uneaten prey mosquitoes may develop into biting adults if left in the cells; and 5) there is a high risk of mosquito obligate infection spreading from the feeders to colonized *Toxorhynchites.*

### Eclosion to L2

Live *Panagrellus redividus* (L.) nematodes. This nematode is mass cultured in plain potato medium (www.Carolina.com, Burlington, NC) and replicates to harvest quantity within 2 wk after subculturing.

**Fig. 7.**
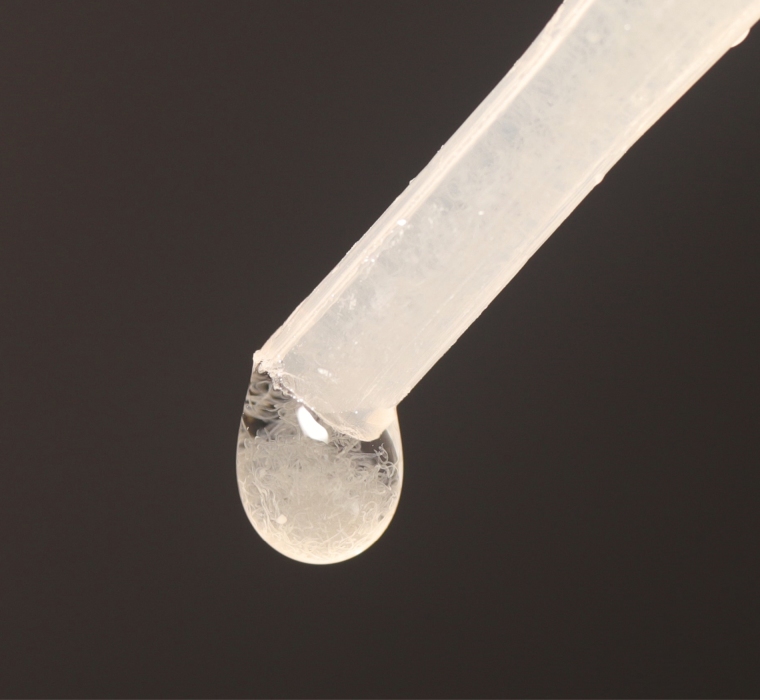
One appropriate-sized drop of *Panagrellus* worms.

**Fig. 8.**
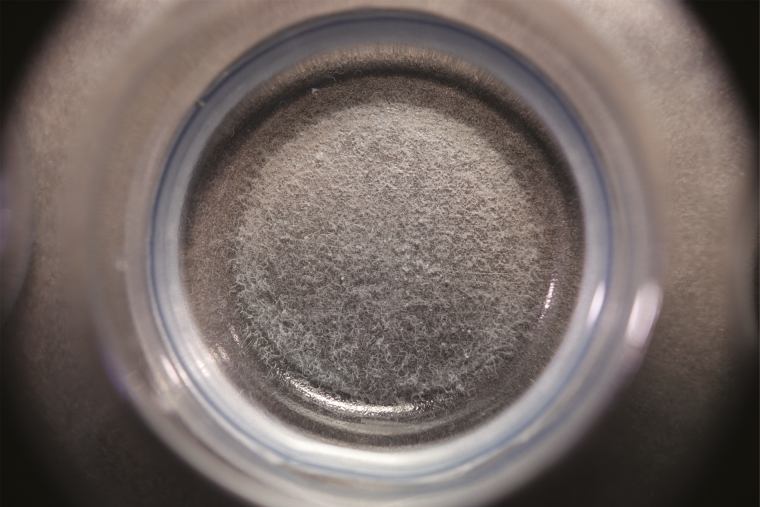
Isolation well with appropriate amount of *Panagrellus* worms in the bottom.

A proven method for harvesting *Panagrellus* worms is by setting up 25-ml Petri dish subcultures from 1-liter mass cultures kept in 2-liter lidded tubs. The tub lids are microperforated to allow gas exchange but small enough to avoid *Panagrellus’* escape. Each Petri dish is filled with ~20 ml of the mass culture spooned off the tubs’ top layer, taking care to leave a remaining *Panagrellus* layer. The lidded Petri dishes are placed at room temperature (74°F/23.3°C) and require a resting period of 10−14 d to allow the worms to migrate to the lid. The number of Petri dishes harvested is based on the number of fresh hatched larvae. One Petri dish lid with abundant worm coverage will feed ~14 L1 larvae. Using a wash bottle filled with RO water, the lids of three to four selected Petri dishes are gently rinsed into an 8-oz collection container. Once the desired quantity is collected, each bowl is filled to the top lower lip with RO water. The worms will settle to the bottom within 1 min or so. The container is slowly decanted to avoid disrupting and spilling the tiny worms. Two to three decanting/refilling cycles over a 10-min period are required to separate the worms from their waste and incidental potato medium. Inadequate rinsing and subsequent introduction of the medium-rich water into the *Tx. rutilus* rearing cell will result in fouling the culture water. Using a 3-ml disposable pipette, the rinsed worms are drawn up and administered by 50-µl drops (Fig. 7). Ideally, the worms are pipetted to the rearing cells before the hatchlings are added. Two full drops will fill the rearing cells with enough worms to feed each larva until the next instar (Fig. 8).

### L2–3

Live culicine or aedine mosquito larvae for food are reared in ½ pans (Table 3) slowly filled halfway with RO water with ¼–½ tsp of ground flake goldfish food added. The pans are set up several hours ahead of time, allowing the water to come to ambient temperature and the fish food to settle to the bottom. For culicine culture, ~100 rafts per ½ pan (Table 3) are rested on top of the water. For *Ae. aegypti or Ae. albopictus,* egg papers encrusted with ~2,000 eggs are submerged. Feeder larvae are drained into a fine screen sieve, rinsed with RO water to remove debris and waste, placed into fresh RO water, and pipetted into each well. Around 10–15 three- to four-day old culicine/aedine larvae can be fed off to each *Tx. rutilus* larva on development day 5 or 6. Uneaten feeder larvae/pupae must be removed after 4 d in the cells else they may emerge as biting adults.

**Table 3.** Container dimensions

Container size (restaurant standards)	Length or radius (cm)	Width (cm)	Depth (cm)	Surface Area (cm^2^)
Full	49.5	29.5	6	1460.3
1/2	29.5	24	6	708.0
1/3	29.5	15	6	442.5
8 oz	5.50	–	4	95.0
2 oz	2.87	–	3.25	25.9
1 oz	2	–	3	12.6

### L2–3 Prey Alternative

Live *Dero* sp. worms (aquatic Annelid, Oligocheata, Naididae). Our in-house *Dero* worm culture was sourced from outdoor garden variety, water storing bromeliads (*Neoregelia* sp., *Guzmania* sp.), where *Tx. rutilus* larvae were observed feeding upon the worms. The *Dero* worms are cultured in shallow, wide surface area containers, filled with no more than 3-cm depth RO water and provided with sinking algae wafer fish pellets as food. A 20- × 20-cm piece of fiberglass window screen with 10- × 10-mm mesh and a 10- × 20-cm piece of unbleached, brown paper are added for substrate. Approximately 500 ml of *Dero* worm culture is adequate for 1,000 *Tx. rutilus* larvae when fed once per week, allowing the worm cultures time to recover and replicate. About 5 ml of worms will feed ca. 100 L3 *Tx. rutilus* larvae to their L4 stage, when thawed bloodworms are added. The *Dero* worms are harvested via pipette or mesh scoop and transferred into a graduated cylinder. Once settled, water is removed to compact worms for an accurate measurement. The worms are then placed into fresh RO water, separated gently with a 3-ml pipette, and 10–15 worms are added into each cell. Feeding live *Dero* worms is advantageous over live mosquitoes, as uneaten worms will not be a problem, whereas uneaten mosquito larvae can pupate and adult mosquitoes emerge. Also, the *Dero* worm husbandry is much simpler than that for mosquitoes since the worms exist exclusively in water in a simple, direct life history, are hermaphroditic, and do not require additional resources. The disadvantage is that *Dero* worm replication cycle is primarily asexual via segmental regrowth and budding and takes ca. 8 wk at 80°F/15.5°C water temperature; thus, many worm culture pans are required to supply sufficient food quantity.

### L4

Store bought, blister packaged bloodworms (chironomid larvae) are the final food the larvae will receive until pupation. The bloodworms are kept frozen until use and thawed in RO water, then rinsed twice to remove guar gum (a packaging byproduct which, if not removed, will foul the rearing water). Using a large 8-ml disposable pipette with the tip enlarged via angle cut, 0.3-g bloodworms (or roughly 7 worms) are given to each *Tx. rutilus* larva, up to three times during the final larval stage for a total of 1.0 g. Provisioning the cells with larger quantities than what a L4 larva can consume within 3–4 days will result in water fouling; thus, multiple feedings are required.

Pupa Selection

Using a simple scoop made of fiberglass window screen, sclerotized pupae are transferred to clean pupal containers with *Melaleuca* infused RO water and placed into their cages.

Care must be taken not to handle young, light colored pupae as they are quite fragile and are easily injured. Crowding in the emergence bowl can increase mortality as the tumbling motion by cohorts can knock actively emerging adults to the side, causing their wings to saturate and ultimately lead to drowning. The selected pupa emergence container should allow a water surface area of no less than 3.8 cm^2^ (0.5 in^2^) per pupa (Table 1). However, the ideal method is not to move pupae from their solitary larval cell to a communal emergence bowl at all, but this is not always feasible.

**Fig. 9.**
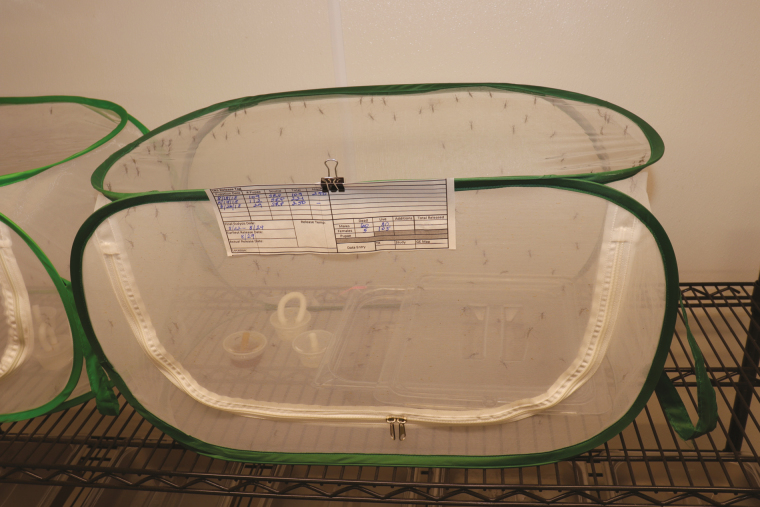
Popup mesh release cage.

Pupa sex cannot be determined morphologically, as is done with other mosquitoes. With *Tx. rutilus* sexes are selected by batch development days, with males pupating 1–2 d before females. Detailed record keeping for each egg batch is necessary to quickly calculate batch development status, thus cages predetermined for release can be set up with an approximately equal gender mix. Bowls with pupae determined for production colony maintenance are either placed directly into the cage or left to emerge in a separate emergence cage and transferred as adults. Pupae destined for release as adults are placed directly into portable 34.29- × 34.29- × 60.96-cm popup mesh cages (Fig. 9; Bioquip, St. Compton, CA; Catalog #1466BV). Emerged adults are fed the honey and sucrose solutions, and water, and kept in the portable cages for 4–6 d to allow mating and oogenesis. An entire release cage is taken to the predetermined study site on the morning of the release day.

### Releases

*Toxorhynchites rutilus* are releasable at any life stage. To increase the likelihood of establishing a new or supplementing a probable population, the habitat should be a favorable match to *Tx. rutilus* biology and include oviposition sites, floral nectar sources, and harborage sites.

Eggs and larvae can be placed directly into and allowed to develop within problem mosquito containers. *Toxorhynchites* spp. eggs are light, loose, and hydrophobic but easily drowned if jostled in water. A safe way to transport them is on a damp paper towel in a lidded vessel. In areas where the container breeding pest mosquito source is unknown or inaccessible, gravid adult females are released in the morning to find the cryptic breeding places themselves. The resulting adults will remain nearby and increase the population further.

## Results and Discussion

The rearing method described was employed to rear 850–1,000 adult *Tx. rutilus* per week for the study releases, experiments and colony maintenance. For the 2017 study alone, roughly 650 adult *Tx. rutilus* were reared weekly and allowed to mate and mature before release. Of those, 300 gravid females were released at multiple select sites (100 per site) for 11 wk, June–September. Their impact was monitored weekly via trapping adult mosquitoes with BG-Sentinel 2 traps (Biogents, AG, Germany) until the end of August when Hurricane Harvey caused widespread flooding, devastated the rearing facility, and resulted in the study’s premature termination. A repeat study is in progress.

We were amazed to observe during the egg eclosion trials that eggs developing in 90°F/32.2°C water and ambient temperatures would not hatch, since mid-summer outdoor temperatures in southeast Texas typically reach into the high 90s°F/ + 35°C, yet we observe wild *Toxorhynchites* during that time. We visited a variety of known outdoor *Toxorhynchites* oviposition sites and took temperature readings at water/egg level and found their temperatures to be in the 80–83.6°F/26.6–28.7°C degree range irrespective of container type.

### Future Possibilities and Obstacles for Large Quantity *Tx. rutilus* Rearing

The development of a nutritionally complete, custom factitious *Tx. rutilus* diet is needed to progress to large-scale production levels. The diet’s formulation must take the effect on water quality into account as *Tx. rutilus* larvae are quite tolerant of hypoxic water conditions but succumb to foul, anoxic rearing water. Further, motion and sensory touch triggers the L1–L3 larva’s feeding response, whereas older L3–L4 larvae will also feed upon nonmotile foods, including detritus. Consequently, if the diet will be offered in the early larval stages, it must be highly attractive in shape and texture to stimulate young larvae’s feeding response.

As with all custom developed entomophagous insect replacement foods, this *Tx. rutilus* larval diet must be selected to produce robust, highly fecund adults for continuous generations. We propose metabolic rate analysis in addition to or instead of conducting full life cycle bioassays. Successful development of an artificial diet replaces the need for careful planning of multiple life cycles and would allow an immense production output, therefore enable the utilization of mechanized and automated feeding techniques.
